# Protecting Our Own: Equity for Employees as Hospitals Battle COVID-19

**DOI:** 10.1089/heq.2020.0024

**Published:** 2020-09-25

**Authors:** Lydia R. Maurer, Numa P. Perez, Emily E. Witt, Gezzer Ortega

**Affiliations:** ^1^ Department of Surgery, Massachusetts General Hospital, Harvard Medical School, Boston, Massachusetts, USA.; ^2^ Harvard Medical School, Boston, Massachusetts, USA.; ^3^ Department of Surgery, Center for Surgery and Public Health, Brigham and Women's Hospital, Harvard Medical School, Boston, Massachusetts, USA.

**Keywords:** COVID-19, Health Equity, social needs, disparities

## Abstract

As medical providers garner praise during the coronavirus disease 2019 (COVID-19) pandemic, “nonclinical” health care workers remain largely overlooked. Although these essential workers face similar, if not greater, risks of contracting severe acute respiratory syndrome coronavirus-2 (SARS-CoV-2) as others on the frontlines, many hospitals have fallen short in providing this vulnerable population with needed protections. Instead, hospitals should implement policies that guarantee all staff receive the information, equipment, and support necessary for battling the current crisis. This is critical not only for promoting the safety of these workers and their families, but also for ensuring the well-being of the community as a whole.

In hospital systems, nonclinical staff members play critical roles but remain under-recognized and increasingly vulnerable. We offer the term “nonclinical healthcare workers” to describe hospital employees often referred to as “support” or “ancillary” staff, including those performing maintenance, janitorial, and patient transport functions, along with food service workers, patient care assistants, and others in roles not directly providing clinical care. We know from the experience in China that the risk of contracting severe acute respiratory syndrome coronavirus-2 (SARS-CoV-2) has been substantially higher for health care workers, with increased exposure and at times inconsistent access to personal protective equipment (PPE).^1^ We also know that in this pandemic, Black and Hispanic populations are dying at higher rates than white populations,^2^ and in the Boston area, for instance, those areas with the highest rates of coronavirus disease 2019 (COVID-19) correspond with lower income neighborhoods.^3^ Unfortunately, many nonclinical health care workers fall into both high-risk groups, in that they have the exposures to COVID-19 patients in their work at the hospital, and fall into lower income brackets (i.e., hospital janitors earn a median of $29,820 a year across the United States).^4^ Overall, individuals from under-represented minority groups are over-represented in frontline service industries, including food service, janitorial staff, housekeeping, and health care support occupations.^5^

The importance of equity in access to health information and care among underserved populations has gained increasing national recognition.^6^ Unfortunately, federal policies to protect nonclinical health care workers specifically are nonexistent, and state-level legislation is limited. In Washington State, the health department did prioritize health care support occupations in the guidelines for allocation of PPE.^7^ However, this type of government protection is limited, and hospitals cannot depend on these types of protections and must take responsibility. Instead, equity must become central to hospital staff policies related to COVID-19. We provide recommendations for hospitals to address critical equity concerns among their employees.

## Be a Source of Truth for COVID-19 Information

Groups with limited English proficiency have historically received inadequate communication of health information during epidemics^8^; the same concern exists during this crisis. Given that many nonclinical health care workers are racial and ethnic minorities,^9,10^ often living in underserved communities at particularly high risk for SARS-CoV-2, hospitals must ensure that accurate culturally appropriate health information is disseminated to these individuals and their families. This information should be appropriate for multiple levels of health literacy, and available in multiple languages. Clear instructions on symptoms and what to do if they get sick are particularly important. Hospital systems should be creative and flexible as they disseminate this information.

Despite hospitals claiming that they have enough PPE for staff and touting communication hotlines and other methods of communication, nonclinical health care workers have reported that messages are often conflicting, confusing, or nonexistent.^11^

In an attempt to address these communication gaps, Mass General Brigham hospital system in Boston, MA, for example, has implemented a multifaceted approach, including text messaging platforms in multiple languages to disseminate timely instructions and reminders, in person dissemination of paper materials, hotlines, and information sessions in multiple languages.^12^ In addition to instituting these interventions, hospital systems should evaluate their effectiveness in the nonclinical health care worker population specifically. There are reports from other health care systems that although hospital administrators think they are providing accessible resources, individuals in the nonclinical health care workforce may still feel like this communication does not reach them.^11^ Providing health care workers from communities highly affected by COVID-19 with accurate and timely information, will empower them to lead local advocacy efforts, and act as a resource and source of peer education and potential cultural broker, which will ultimately help to dispel myths and disseminate truth.

## Provide Equitable Access to PPE and Testing

News reports^11^ have highlighted inconsistent access to PPE for janitorial staff, compared with doctors and nurses. On the CDC website, there is no guidance for hospitals on appropriate use of PPE among nonclinical health care workers,^13^ placing the onus on them to establish their own equitable policies to protect these individuals. Directives for all nonclinical health care workers coming into contact with COVID-19 patients should mirror those going out to doctors and nurses, with instructions specific to the different roles they play. One example of this approach being implemented was at Kaiser Permanente Downy Medical Center, in which unionized workers worked with administration to ensure more protection for environmental service workers who clean rooms between patients (the majority of whom are Latino and many of whom are not fluent in English), including getting access to N95s, gowns, gloves, and booties for these workers.^14^ Training for the use of PPE must be accessible, language appropriate, and culturally sensitive to protect our employees from different backgrounds.

Given that supply of PPE can be limited, creative solutions are sometimes needed to keep workers safe. At Stanford Hospital, members of United Health care Workers West worked with hospital leadership and food service managers to increase protections for dishwashing staff by converting all food service equipment to disposable paper meal service, in addition to providing the appropriate safety equipment for their staff.^14^ Working to guarantee adequate protection for every employee will help hospitals maintain trust with the workers whose efforts keep all of us safe.

Moreover, as testing becomes more widely available, it will be important to ensure that nonclinical health care workers gain equal access to such testing. Some states have recently expanded testing to all essential workers and hospitals should follow suit.^15,16^ Equitable testing policies will also help prevent these employees from spreading the virus among their home communities, which are particularly vulnerable to COVID-19.

## Recognize and Support Increased Social Needs

Even for those nonclinical health care workers who stay healthy, social needs such as housing, food security, transportation, and other financial needs can be real concerns. Linking employees with local resources to address these increased social needs is critical. When employees get sick, clear and well-disseminated plans that account for diverse social situations are vital. In many cases, employees may live in small crowded, multigenerational homes where self-isolation is not possible. Support with alternative housing (e.g., using local hotels),^17^ affordable or free child care options, community-based access to testing for family members, and paid leave are critical to curbing the spread of disease in families and the communities that are already at the highest risk.

Hazard pay has been featured prominently in the news media^18^ as a potential solution to mitigate the risk for health care workers caring for COVID-19 patients. Although it does provide some benefit to the workers who stay healthy, sick leave policies and health insurance coverage for family members who get sick are even more important. The Families First Coronavirus Response Act (FFCRA)^19^ requires employers with 50–500 employees to provide paid sick leave and paid extended family and medical leave for employees. However, hospitals with >500 employees are not compelled to follow this. These hospitals must put in place protocols that encourage and enable sick employees to stay home; otherwise, sick employees may feel compelled to continue working to support their families.

Nonclinical health care workers have always been invisible. Now, they are even more so, as extensive news media coverage emphasizes—appropriately—the risks and sacrifices of doctors and nurses. However, it is critical that we bring the needs of nonclinical health care workers into the forefront. For not only are equitable policies important to keep these employees and their families safe, but they also play a vital role in ensuring the safety of our patients and the community at large.

## Abbreviations Used

COVID-19: coronavirus disease 2019
FFCRTA: Families First Coronavirus Response Act
PPE: personal protective equipment
SARS-CoV-2: severe acute respiratory syndrome coronavirus-2
